# Diversity and antibiotic resistance of cultivable bacteria in bulk tank milk from dairy farms in Shandong Province, China

**DOI:** 10.3389/fvets.2025.1649876

**Published:** 2025-07-23

**Authors:** Yijian Qi, Zhiyuan Lu, Ziru Meng, Xiaozhou Wang, Huahua Chen, Muzi Li, Chaonan Qu, Pu Zhang, Yongxia Liu, Jianzhu Liu

**Affiliations:** ^1^College of Veterinary Medicine, Shandong Agricultural University, Tai’an, Shandong, China; ^2^Shandong Provincial Key Laboratory of Zoonoses, Shandong Agricultural University, Tai’an, China; ^3^College of Life Sciences, Shandong Agricultural University, Tai'an, China; ^4^The Affiliated Tai’an City Central Hospital of Qingdao University, Tai’an, China

**Keywords:** bacterial diversity, antibiotic resistance, bulk tank milk, dairy farm, antibiotic resistance genes

## Abstract

**Introduction:**

This study systematically analyzed bacterial diversity and antimicrobial resistance (AMR) profiles in bulk tank milk from five dairy farms (*n* = 30) in Shandong Province, China, to assess public health risks associated with microbial contamination and provide critical data for regional quality control and AMR risk assessment in dairy production systems.

**Methods:**

Total bacterial counts were quantified, revealing significant inter-farm variation (*P* < 0.05) with a range of 3.94–6.68 log CFU/mL. Among 129 bacterial isolates, genus-level dominance and species prevalence were identified. Antimicrobial susceptibility testing (AST) against 10 agents was performed using integrated resistance criteria combining Clinical and Laboratory Standards Institute (CLSI) standards and epidemiological cutoff values (ECOFFs). Nine resistance genes targeting seven antibiotic classes were detected via PCR.

**Results:**

The highest resistance rate was observed for sulfadiazine (53.2%) and the lowest for levofloxacin (6.0%). Multidrug resistance was detected in 23% (20/87) of isolates, with 14 strains meeting ECOFFs-based resistance criteria. PCR analysis showed *sul1* (70.5%) and *ant(4′)-Ia* (54.3%) as the most prevalent resistance genes, while *mcr-1*, *lnu* (B), and *bla*_NDM-1_ were absent in all isolates. Regional resistance variations correlated significantly with farm management practices.

**Discussion:**

These findings underscore the impact of historical antibiotic use on AMR dissemination. Enhanced AMR surveillance in raw milk, improved antibiotic stewardship, and targeted interventions are crucial to mitigate public health risks from microbial contamination and horizontal gene transfer of resistance determinants.

## 1 Introduction

Raw milk acts as a critical vector for microbial contamination due to its physicochemical properties, with bacterial loads directly compromising dairy safety (1). The presence of spoilage and pathogenic bacteria in bulk tank milk poses significant risks to public health (2). Cattle ingesting contaminated forage introduce bacteria into their systems, where they proliferate and disseminate into the environment via feces (3). Total bacterial count (TBC) in raw milk is predominantly influenced by sanitation practices. For instance, Sun et al. (4) demonstrated that farms implementing chemical teat disinfection protocols with regular acid washes achieved significantly reduced TBC levels. Contamination by genera such as *Pseudomonas*, *Acinetobacter*, *Bacillus*, *Micrococcus*, *Enterobacter*, *Enterococcus*, and *Aerococcus* critically threatens dairy quality (5). Notably, thermoduric microorganisms like *Bacillus licheniformis* frequently detected in dairy powders as well as *Bacillus cereus* and *Bacillus subtilis*, are key contributors to milk rancidity (6, 7). Conventional microbial indicators (e.g., *Streptococcus*, *Staphylococcus*, coliforms) remain vital for safety evaluation (8). Although the issue of microbial contamination in dairy products has been extensively studied, in the important dairy production base of Shandong Province in China, the relationship between the bacterial diversity in raw milk and the spread of antibiotic resistance, especially the relationship with farm management practices, is still poorly understood.

The escalating use of antimicrobials in livestock management has driven the emergence of multidrug-resistant (MDR) pathogens, critically jeopardizing dairy safety (9). Bastam et al. reported contamination rates of 60% for *Staphylococcus aureus* (*S. aureus*) and 53% for *Salmonella enterica* serovar Typhi (*S. typhi*) in dairy samples, underscoring the urgency of antimicrobial resistance (AMR) containment (10). Alarmingly, rising resistance to last-resort antibiotics (e.g., carbapenems, cephalosporins) further restricts therapeutic options (11). In China, the National Food Safety Standard (GB 31650.1-2022) establishes maximum residue limits (MRLs) for veterinary antibiotics in raw milk, including *β*-lactams (penicillin G: 4 μg/kg), tetracyclines (oxytetracycline: 100 μg/kg), and sulfonamides (sulfadiazine: 25 μg/kg). However, small-scale and backyard dairy operations exhibit a 44% non-compliance rate with antibiotic withdrawal periods, largely due to poor medication record-keeping (12). Inadequate milking equipment sanitation further exacerbates cross-contamination risks, leading to antibiotic residues exceeding regulatory thresholds within single milk batches (13). Indiscriminate antibiotic use not only amplifies AMR in livestock pathogens but also facilitates horizontal transfer of antibiotic resistance genes (ARGs) to human-associated bacteria via the food chain, posing dual threats to animal welfare and public health (14, 15).

The persistence of antibiotic resistance genes (ARGs) in environmental bacteria (16), such as Pseudomonas and Acinetobacter, is facilitated by mobile genetic elements that enable horizontal gene transfer (HGT) across phylogenetically distinct species. This dynamic interaction within diverse microbial communities may accelerate the evolution of multidrug-resistant strains, particularly in settings with suboptimal sanitation practices. For decades, antibiotics were routinely added to animal feed not only for therapeutic purposes but also as growth promoters, and such historical practices of overuse have directly contributed to the proliferation of ARGs (17). Although antimicrobial growth promoters have been banned in the European Union since 2006 and restricted in many countries worldwide, with China having significantly reduced their usage in recent years, the legacy of prolonged and extensive antibiotic exposure remains a key driver of persistent ARG dissemination in environmental settings. This abuse leads to ARGs being spread into the ecosystem through husbandry manure, causing environmental pollution and perpetuating the cycle of resistance (18, 19). ARG contamination now poses a critical global health challenge, demanding urgent mitigation strategies.

However, although microbial contamination in dairy products has been extensively studied, in the important dairy production base of Shandong Province, China, the relationship between bacterial diversity in raw milk and the spread of antibiotic resistance, particularly its linkage to regional farm management practices, remains poorly understood. This study aims to systematically analyze the bacterial diversity and antimicrobial resistance profiles in bulk tank milk from Shandong dairy farms. By integrating farm management data, we seek to evaluate the role of environmental bacteria as reservoirs of ARGs and their potential contribution to resistance dissemination, thereby informing region-specific interventions for dairy safety.

## 2 Materials and methods

### 2.1 Sample collection

From April to June 2022, 30 raw cow milk samples were systematically collected from 30 bulk milk tanks in five dairy farms (six samples per farm) across three different cities of Shandong Province (one farm from Taian City [TA], two farms from Dongying City [DY], and two farms from Qingdao City [QD]). Each sample was collected through aseptic mixing of raw cow milk obtained from multiple spatially distributed areas within the same bulk milk tank. All the combined samples were placed in 10-ml sterile centrifuge tubes and stored at 4°C and then transported to the laboratory for further analysis. Through the on-site epidemic prevention supervisor and the farm records, the disinfection procedures of the site, the usage of antibiotics, and the operation problems of the equipment were obtained. All experimental procedures complied with the guidelines of the Animal Welfare and Ethics Committee at Shandong Agricultural University and received approval (SDAU2022-00168).

### 2.2 Enumeration of bacteria

The sample was subjected to ten-fold serial dilutions in Stroke-physiological Saline Solution (SPSS) (Beyotime Biotechnology Co., Ltd., Shanghai, China), achieving a final dilution of 10^−2^. Subsequently, 5 μL aliquots from each dilution were inoculated onto Brain Heart Infusion agar (Beijing Solarbio Science & Technology Co., Ltd., Qingdao, China) supplemented with 5% sheep blood (BHI-blood agar) and incubated aerobically at 37°C for 24 h. Plates containing from 30 to 300 colonies were counted. Results were expressed as Colony-forming units per milliliter (CFU/ml) and were converted to log (CFU/ml).

### 2.3 Isolation and identification of bacteria

On BHI blood agar plates, according to differences in colony diameter, shape (round, irregular, filamentous), color (white, cream, yellow), surface texture (smooth, rough, slimy), edge features (smooth, wavy), and hemolysis patterns (*β*-hemolysis, *α*-hemolysis, non-hemolytic), all representative colony types showing distinguishable morphological differences were selected. And under the same culture conditions, these were repeatedly streaked onto BHI blood agar plates. This process was continued for 2–3 times, until each isolate obtained a pure culture exhibiting single colony morphology. Using an inoculating loop, pick a single colony and transfer it to a sterile centrifuge tube containing BHI broth, followed by incubation under appropriate conditions for a defined duration. Subsequently, add 20% glycerol to the culture, vortex thoroughly for homogenization, and store at −80°C for future use. Bacterial identification was performed using a matrix-assisted laser desorption/ionization time-of-flight mass spectrometer, (MALDI-TOF MS) (MALDI Biotyper, Bruker, Bremen, Germany) following the manufacturer’s standard operating protocols.

### 2.4 Antimicrobial susceptibility testing

Drug susceptibility testing was performed according to the broth microdilution, recommended by the Clinical and Laboratory Standard Institute (CLSI) (20). Two-fold serial dilutions of each antibiotic were prepared in 96-well plates (Costa) using appropriate media selected based on bacterial species, with concentration ranges spanning from 0.125 to 512 μg/mL. Incubation periods vary depending on species: 16–20 h for *Enterobacteriaceae* and *Pseudomonas*, while 20–24 h for *Staphylococcus*, *Enterococcus*, *Acinetobacter*, and *Streptococcus*. *Escherichia coli* (*E. coli*) ATCC 25922 and *S. aureus* ATCC 29213 were used as quality control strains. The tested panel included ten antimicrobial agents spanning seven classes of antibiotics: ceftriaxone sodium (CRO), penicillin (PCN), levofloxacin (LVX), sulfadiazine (SDZ), lincomycin (LIN), colistin sulfate (CLS), gentamicin (GM), doxycycline (DOX), neomycin (NEO), and streptomycin (SM). All the above drugs were purchased from the Shanghai MacLean Company (Shanghai, China). The result interpretation time was experimentally observed to vary significantly across bacterial species. The MIC was defined as the lowest drug concentration demonstrating complete inhibition of visible bacterial growth in the test wells. The measured MIC values were compared against the clinical breakpoint (CBP) standards established by the CLSI (CLSI M100 35th ed., 2025) and the European Committee on Antimicrobial Susceptibility Testing (EUCAST, v 12.0 Breakpoint Tables) (20, 21). In cases where CBPs for specific antimicrobial agents are not defined by CLSI or EUCAST, an integrated assessment utilizing epidemiological cutoff values (ECVs [CLSI terminology]/ECOFFs [EUCAST terminology], where resistance was presumptively classified based on non-wild type (NWT) status) and resistance gene detection should be implemented (21, 22). Isolates exhibiting MICs exceeding the ECOFF and demonstrating the presence of a resistance-associated gene known to confer resistance to the specific antimicrobial agent were provisionally classified as candidate resistant strains. MDR bacteria are defined as those that are resistant to at least one antimicrobial agent in each of three or more antimicrobial classes (23).

### 2.5 DNA extraction

The boiling method was used to extract DNA (24). Single colonies were picked and incubated to the logarithmic phase at 37°C, 200 rpm in Luria-Bertani (LB) broth medium. The bacterial culture was centrifuged at 12,000 × g for 30 s at 4°C to obtain cellular precipitates. The precipitates were mixed with Tris-EDTA (TE) buffer (Thermo Fisher Scientific Co., Ltd., Shanghai, China) and subjected to cellular disruption by boiling at 98°C for 10–15 min. The tubes were immediately placed on ice for ten minutes and then centrifuged at 13,000 rpm for five minutes to collect the clarified supernatant, thereby harvesting DNA from each isolate. The collected samples were stored at −20°C until further processing.

### 2.6 Screening for antibiotic resistance genes

The 129 isolates were screened for the presence of nine antibiotic resistance genes by PCR, including *β*-lactam resistance genes (*bla*_KPC_ and *bla*_NDM-1_), sulfonamide resistance gene (*sul1*), tetracycline resistance gene (*tet*(M)), aminoglycoside resistance genes (*ant(4′)-Ia* and *aph(2″)-Ic*), quinolone resistance gene (*qnrS*), lincosamide resistance gene (*lnu*(B)), and polymyxin resistance genes (*mcr-1*). The PCR was performed with 25 μL volumes composed of 12.5 μL of 2 × Taq Master MIX (Vazyme Biotech Co., Ltd., Nanjing, China), 2 μL of primers, 1 μL of 100 ng of DNA template, and 9.5 μL DEPC-Treated Water (NCM Biotech Co., Ltd., Suzhou, China). All primers are listed in Table 1.

**Table 1:** Primer sequences used in the study.

Gene	Primers (5′ to 3′)	Length (bp)	Reference
*bla*_NDM-1_	F: GTCTGGCAGACTTCCTATCTC R: GGTTCGACAACGCATTGGCATAAG	268	Wang et al. (25)
*bla*_KPC_	F: CGTCTAGTTCTGCTGTCTTG R: CTTGTCATCCTTGTTAGGCG	798	Poirel et al. (26)
*lnu*(B)	F: CCTACCTATTGTTTGTGGAA R: ATAACGTTACTCTCCTATTC	925	Stepien-Pysniak et al. (27)
*tet*(M)	F: GTGGACAAAGGTACAACGAG R: CGGTAAAGTTCGTCACACAC	406	Stepien-Pysniak et al. (27)
*ant(4′)-Ia*	F: CAAACTGCTAAATCGGTAGAAGCC R: GGAAAGTTGACCAGACATTACGAACT	294	Stepien-Pysniak et al. (27)
*aph(2′′)-Ic*	F: CCACAATGATAATGACTCAGTTCCC R: CCACAGCTTCCGATAGCAAGAG	444	Stepien-Pysniak et al. (27)
*sul1*	F: TTCGGCATTCTGAATCTCAC R: ATGATCTAACCCTCGGTCTC	822	Chaturvedi et al. (28)
*qnrS*	F: ACGACATTCGTCAACTGGAA R: TTAATTGGCACCCTGTAGGC	417	Doma et al. (29)
*mcr-1*	F: CGGTCAGTCCGTTTGTTC R: CTTGGTCGGTCTGTAGGG	309	Li et al. (30)

### 2.7 Statistical analysis

Data organization was completed using Microsoft Excel 2021 (Microsoft Corporation, Redmond, WA, United States), and graphs were generated using Origin 2022 (Origin Lab Corporation, Northampton, MA, United States). The data are expressed as means ± SD. Statistical significance was determined based on one-way analysis of variance (ANOVA) in appropriate conditions using GraphPad Prism 8 software. Significance was determined at *p* < 0.05.

## 3 Results

### 3.1 Total bacteria count in raw milk samples

**Figure 1:**
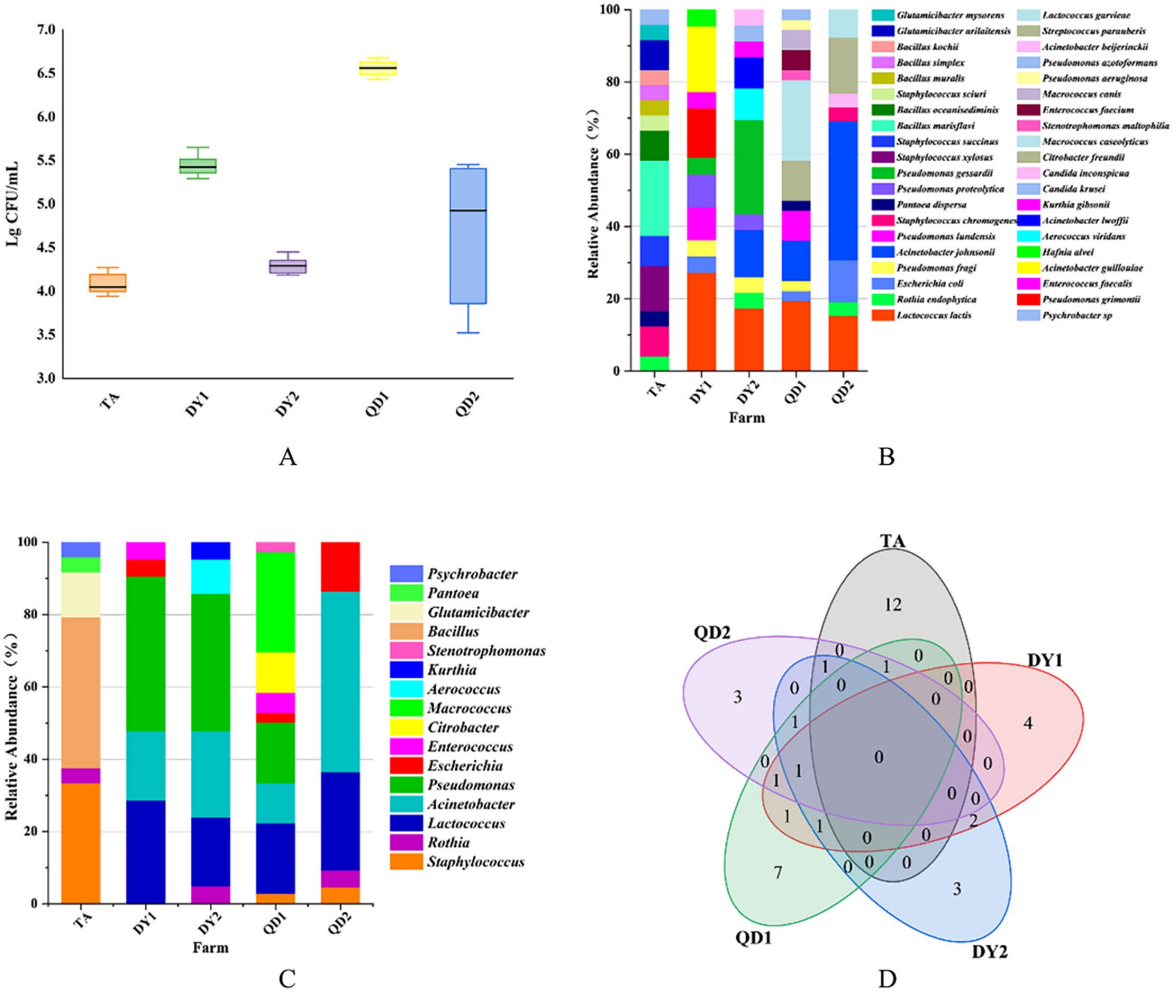
Total bacterial count (TBC) from Taian (TA), Dongying1 (DY1), Dongying2 (DY2), Qingdao1 (QD1) and Qingdao2 (QD2) dairy farms **(A)**. Relative abundance of bacterial species **(B)** and family **(C)** from these dairy farms. Venn diagram of correlation of bacterial species in different dairy farms **(D)**.

The TBC of raw cow milk samples in five dairy farms ranged from 3.94 to 6.68 log CFU/ml (Figure 1A). Significant inter-farm variation was observed (*p* < 0.05). QD1 dairy farm had the highest TBC, with values ranging from 6.43 to 6.68 log CFU/ml, while TA dairy farm had the lowest TBC, ranging from 3.94 to 4.27 log CFU/ml. This discrepancy may be attributed to differences in equipment sanitation protocols across farms. Although all farms implemented standardized chemical disinfection protocols before and after daily operations, TA dairy farm uniquely adopted supplementary thermal disinfection (≥85°C for 15 min) followed by thorough drying for heat-resistant components of milk storage tanks. In contrast, DY2 and QD1 dairy farms showed suboptimal cooling system efficiency in their storage tanks, potentially contributing to prolonged microbial proliferation. Overall, TBC in DY2 and QD1 dairy farms were higher than that in other dairy farms.

### 3.2 Identification of bacteria

Matrix-assisted laser desorption/ionization time-of-flight mass spectrometry (MALDI-TOF MS) identified 129 isolates spanning 37 species and 13 families. Notably, pathogenic bacteria associated with bovine mastitis (*Staphylococcus xylosus, Staphylococcus chromogenes, Streptococcus parauberis*) were isolated, accounting for 8.53% (11/129) of the total isolates. Spoilage-related bacteria (*Pseudomonas lundensis, Pseudomonas proteolytica, Pseudomonas gessardii, Pseudomonas fragi*) constituted 13.95% (18/129), while opportunistic pathogens (*Acinetobacter lwoffii, Acinetobacter johnsonii, E. coli*) represented 20.16% (26/129) of the identified microbiota (Table 2). Geographically, TA farm harbored 14 bacterial species, with *Bacillus marisflavi* as the predominant species. The pathogenic bacteria belonging to the *Staphylococcus* genus accounted for 20.8% (5/24) (*p* < 0.05; Figure 1B). DY1 and DY2 farms yielded 10 and 9 species, respectively, dominated by *Lactococcus lactis* (DY1) and *Pseudomonas gessardii* (DY2) (*p* < 0.05). DY1 farm exhibited a notably high prevalence of spoilage-related bacteria 13.6% (3/22), followed by opportunistic pathogens 22.7% (5/22). In contrast, DY2 farm demonstrated comparable microbial patterns, with spoilage-related bacteria constituting 38.1% (8/21) and opportunistic pathogens accounting for 38.1% (8/21). QD1 farm exhibited 13 species, primarily *Macrococcus caseolyticus*, while QD2 farm isolated 8 species, dominated by *Acinetobacter johnsonii* (*p* < 0.05). Notably, spoilage-related bacteria constituted 16.7% (6/36) and opportunistic pathogens represented 25% (9/36) of the microbiota in QD1. In contrast, QD2 showed markedly lower proportions of spoilage-related bacteria 3.8% (1/26) and opportunistic pathogens 15.4% (4/26), but exhibited a higher prevalence of mastitis-associated pathogens 23.1% (6/26). At the family level, TA isolates (*n* = 24) belonged to 6 families, predominantly *Bacillus*. DY1/DY2 farms each contained 6 families, with *Pseudomonas* as the dominant taxon. In QD, QD1 and QD2 farms harbored 7 and 5 families, dominated by *Macrococcus* and *Acinetobacter*, respectively (Figure 1C). Notably, *A. johnsonii*, *E. coli*, *Lactococcus lactis*, *Pseudomonas fragi*, *Pseudomonas lundensis*, and *Rothia endophytica* were exclusively detected in DY/QD farms, absent in TA (Figure 1D).

**Table 2:** Bacterial isolation and identification across sampled dairy farms.

Dairy farm	Total isolated bacteria	Spoilage-related bacteria (%)	Pathogenic bacteria (%)	Opportunistic pathogens (%)	Others (%)
TA	24	0% (0/24)	20.8% (5/24)	0% (0/24)	79.2% (19/24)
DY1	22	13.6% (3/22)	0% (0/22)	22.7% (5/22)	63.6% (14/22)
DY2	21	38.1% (8/21)	0% (0/21)	38.1% (8/21)	23.8% (5/21)
QD1	36	16.7% (6/36)	0% (0/36)	25% (9/36)	58.3% (21/36)
QD2	26	3.8% (1/26)	23.1% (6/26)	15.4% (4/26)	57.7% (15/26)

### 3.3 Antimicrobial resistance

**Figure 2:**
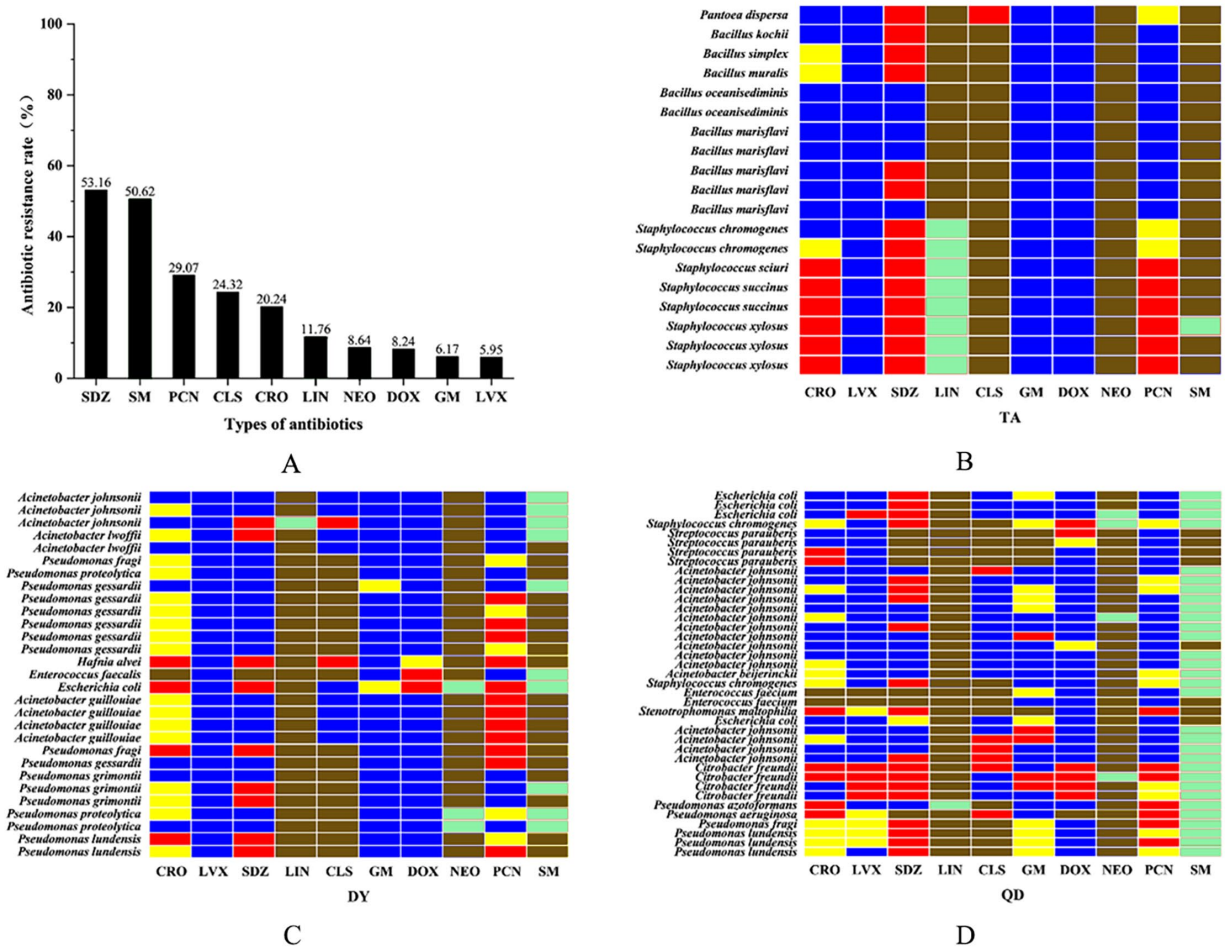
The overall resistance rate of the isolated bacteria to ten antibiotics. Ceftriaxone sodium (CRO), penicillin (PCN), levofloxacin (LVX), sulfadiazine (SDZ), lincomycin (LIN), colistin sulfate (CLS), gentamicin (GM), doxycycline (DOX), neomycin (NEO), and streptomycin (SM) **(A)**. Isolated heat resistance spectrum in Taian (TA), Dongying (DY) and Qingdao (QD) cities. Red: resistant/Blue: susceptible/Yellow: intermediate/Green: non-wild type (NWT)/Brown: clinical breakpoint not established **(B–D)**.

**Figure 3:**
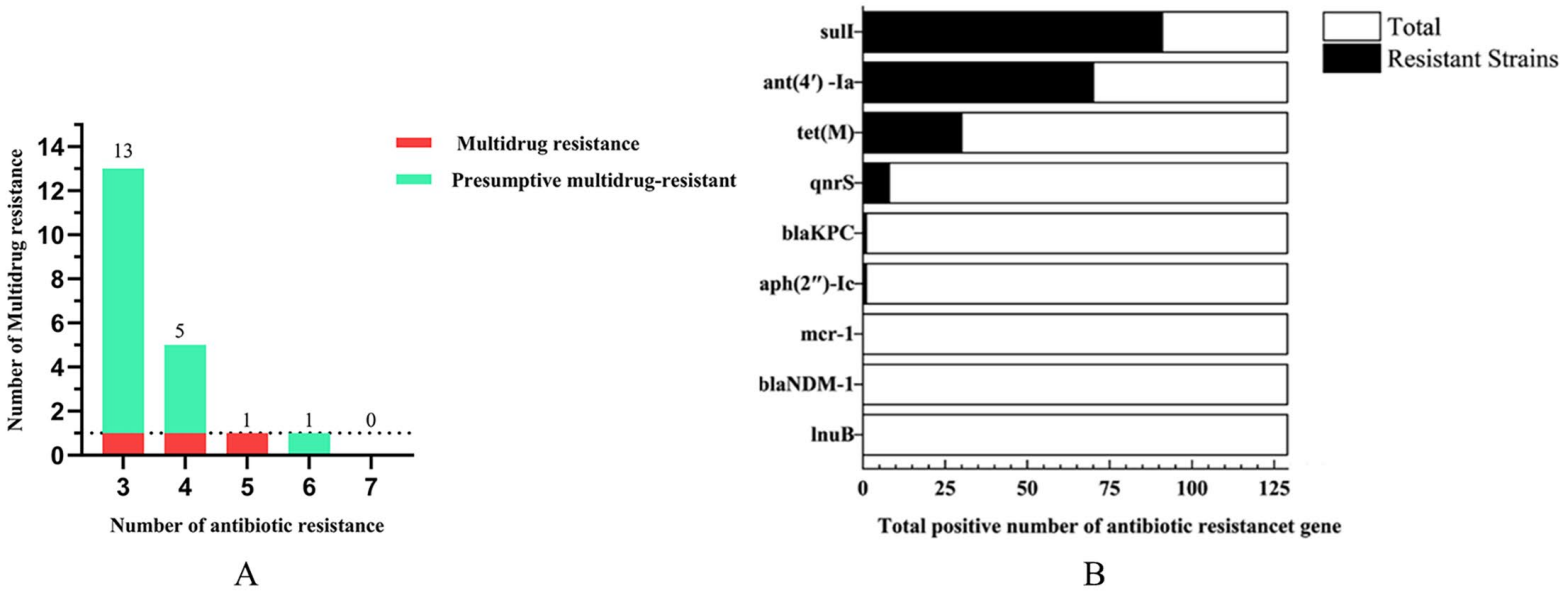
The number of multidrug resistance **(A)** and antibiotic resistance genes **(B)**.

A representative subset of 87 bacterial strains encompassing all identified species/genera was selected from 129 isolates. Antimicrobial susceptibility testing (AST) against ten agents from seven antibiotic classes (with intermediate categorized as non-resistant) revealed significant inter-agent resistance heterogeneity. For isolates lacking established clinical breakpoints for specific antibiotics, data were excluded from final resistance rate calculations (Figures 2A–D). SDZ exhibited the highest resistance rate 53.16% (42/79), with marked regional disparities: TA and DY isolates showed high PCN resistance, whereas QD isolates remained susceptible. Region-specific resistance profiling further demonstrated that: TA isolates exhibited prevalent resistance to CRO, SDZ, and PCN, with mastitis-associated *S. xylosus* showing strong resistance to CRO, SDZ, and PCN, and *S. chromogenes* displaying high resistance to SDZ, and PCN; DY isolates displayed elevated resistance to SDZ, PCN, and SM, notably with opportunistic *A. johnsonii* showing SM resistance and *E. coli* resistant to CRO, SDZ, DOX, PCN, and SM; QD isolates were predominantly resistant to SDZ and SM, where both mastitis-associated *S. chromogenes* and spoilage-related *P. lundensis* exhibited strong dual resistance to these agents. Among the tested isolates, only those originating from QD exhibited 50.6% resistance to SM (42/83), as determined by exceeding ECOFFs and confirming the presence of resistance associated genetic determinants in the absence of clinical breakpoints. Within the 23% (20/87) MDR isolates identified (Figure 3A), 70% (14/20) were classified as MDR candidates based on resistance to at least three antimicrobial classes using this integrative assessment. This resistance landscape suggests potential HGT of resistance determinants from environmental commensals to pathogens via mobile genetic elements, particularly in farms with suboptimal hygiene conditions where complex microbial communities provide ecological niches for HGT-driven MDR evolution.

### 3.4 Antimicrobial resistance gene

Genomic analysis of 129 isolates revealed nine ARGs spanning seven drug classes: sulfonamide (*sul1*), aminoglycosides [*ant(4′)-Ia, aph(2″)-Ic*], tetracycline (*tet*(M)), quinolone (*qnrS*), *β*-lactam (*bla*_KPC_*, bla*_NDM-1_), polymyxin (*mcr-1*), and lincosamide (*lnu*(B)). The detection rate of *sul1* (70.5%) surpassed other ARGs, followed by *ant (4′)-Ia* (54.3%), while *mcr-1*, *bla*_NDM-1_, and *lnu*(B) were absent in all isolates (Figure 3B). Geographically, *sul1* (70.8%), *ant(4′)-Ia* (54.2%), and *tet*(M) (54.2%) dominated TA isolates; DY isolates exhibited higher *ant(4′)-Ia* (74.4%) and *sul1* (60.5%); QD isolates showed *sul1* (63.5%) and *ant(4′)-Ia* (44.5%) prevalence (Table 3). Notably, the ARG distribution demonstrated concordance with regional resistance phenotypes. In TA, the high prevalence of *sul1* (70.8%) correlated strongly with observed SDZ resistance (73.7%). In DY, elevated *ant(4′)-Ia* detection (74.4%) aligned with high SM resistance across tested isolates. For QD, the co-occurrence of *sul1* (63.5%) and *ant(4′)-Ia* (44.5%) paralleled the dual SDZ/SM resistance patterns identified in both pathogenic and spoilage-related bacterial populations.

**Table 3:** The positive rate of antibiotic resistance genes in different cities.

Antibiotic resistance genes	The number of genes in different cities			The percent of genes in different cities		
	Taian	Dongying	Qingdao	Taian	Dongying	Qingdao
*bla*_NDM-1_	0	0	0	0	0	0
*bla*_KPC_	0	0	2	0	0	3.2%
*lnu*(B)	0	0	0	0	0	0
*tet*(M)	13	7	10	54.2%	16.3%	15.9%
*ant(4′)-Ia*	13	32	28	54.2%	74.4%	44.4%
*aph(2′′)-Ic*	0	0	1	0	0	1.6%
*sul1*	17	26	40	70.8%	60.5%	63.5%
*qnrS*	4	1	4	16.7%	2.3%	6.3%
*mcr-1*	0	0	0	0	0	0

The congruence between resistance genotypes and phenotypes not only confirms the functional validity of ARGs but also elucidates potential dissemination pathways traversing environmental reservoirs, commensal microbiota, and pathogenic communities. These findings collectively emphasize the imperative for multidimensional containment strategies targeting critical junctions of antimicrobial resistance transmission networks.

## 4 Discussion

The dairy farm environment harbors diverse bacterial species, including opportunistic pathogens (e.g., *Klebsiella pneumoniae* (25)), foodborne pathogens (*S. aureus* (26), *Campylobacter* (27)), and toxin-producing strains. These bacteria can be directly transmitted through raw milk or enter the dairy product chain through fecal contamination (28). Microbial contamination in bulk tank milk poses significant food safety risks. While enterotoxigenic *S. aureus* was not detected here, its documented role in dairy-associated poisoning underscores contamination hazards. MDR *Campylobacter* and *Salmonella* can spread through raw milk, making post-infection treatment difficult (29, 30). The complex microbial community in raw milk also provides an environment for the transfer of ARGs. For instance, *Enterococcus* can transfer the *tet*(M) gene to *Lactobacillus* spp. through conjugation. Cross-contamination facilitates metabolic exchanges among different bacterial communities and accelerates the spread of ARGs (28).

The microbial diversity in raw milk is profoundly influenced by farm hygiene practices (31, 32). TA farm’s low TBC (3.94 log CFU/mL) and limited MDR isolates correlates with its rigorous thermal disinfection protocols, which reduce bacterial load and potentially limit HGT by disrupting biofilms. Regional variations also stem from environmental adaptation (33): QD farms’ inadequate cooling systems promoted psychrotrophic Pseudomonas growth (Figure 1C), which carried *sul1* (60.5%) and acted as ARG reservoirs (34). Optimizing sanitation protocols could disrupt these ecological niches, thereby curbing HGT-driven resistance dissemination (35).

AST revealed alarming resistance rates to SDZ (53.2%) and SM (50.6%) (Figure 2A), consistent with historical overuse of first-line antibiotics in livestock (36). Through interviews with the on-site epidemic prevention supervisor and reviews of farm records, we found that the high SDZ resistance correlated with its routine prophylactic use against mastitis and respiratory infections in sampled farms. In QD farms, inadequate resting space for cows increased the risk of udder injury. Combined with inconsistent feed sourcing and underdeveloped disease prevention systems, this led to reliance on SM and CLS for infection control. This explains the exclusive detection of SM resistance (50.6%) in QD isolates and elevated CLS resistance (24.3%). In contrast, TA and DY farms exhibited no recent SM usage, highlighting regional disparities in antibiotic practices. Integration of ECOFFs and PCR identified 23.0% (20/87) of isolates as MDR/presumptive MDR strains (Figure 3A).

However, certain limitations should be acknowledged. This approach may overlook novel resistance mechanisms and is limited by ECOFF variability across bacterial populations. Conducting a single sampling study only on dairy farms within Shandong Province will limit its general applicability in a larger geographical area and will not be able to accurately assess the dynamics of resistance evolution. Relying on culturable bacteria for screening high-priority genes may underestimate the overall microbial diversity, resulting in an inability to comprehensively evaluate the complete set of resistance genes.

Regional resistance patterns mirrored global trends, with QD’s SM resistance comparable to Turkish dairy isolates (37); while penicillin (PCN) resistance (48%) was lower than Iranian reports (38). Notably, 20.7% of isolates exhibited resistance to 3–4 drug classes, underscoring urgent needs for targeted interventions. Crucially, even partial compliance with antibiotic restrictions can mitigate resistance emergence, but requires synergism with hygiene improvements to block HGT pathways. The dominance of sul1 (70.5%) and *tet*(M) (54.2%) in environmental bacteria aligns with historical sulfonamide/tetracycline applications in livestock. Selective pressure from prolonged antibiotic use likely enriched these ARGs in bacterial populations (39), facilitating HGT via mobile genetic elements such as plasmids and integrons (40). DY farms’ high *ant(4′)*-Ia prevalence (74.4%) in Pseudomonas may enable aminoglycoside resistance transfer to co-existing pathogens like *E. coli*, particularly under suboptimal sanitation conditions that promote mixed microbial communities. Previous studies have monitored the changes in the pool of AMR genes in raw milk and cheese from farm to consumer (41).

In this study, we did not detect the specific genes such as *mcr-1*, *lnu*(B) and *bla*_NDM-1_. This result may be influenced by various factors, including regulatory restrictions, sampling scope, geographical differences, and the specific bacterial populations present in the sampled milk tanks. However, the persistence of the sulfonamide resistance gene *sul1* and the tetracycline resistance gene *tet*(M) indicates the tenacity of horizontal transferable resistance determinants, emphasizing the importance of continuous vigilance and providing valuable insights for public health monitoring. While our data are specific to cow milk, the detected AMR genes have been similarly reported in small ruminant and buffalo dairy systems under intensive farming conditions (42, 43).

In conclusion, our findings demonstrate the interconnected relationships among microbial diversity, antibiotic, and ARG dissemination within dairy farming systems in Shandong Province. The systematic integration of TBC metrics, AMR profiles, and ARG distribution patterns provides actionable strategies for reducing AMR risks. These evidence-based insights establish a critical foundation for developing targeted intervention protocols and implementing genomic surveillance of multidrug-resistant pathogens, thereby mitigating AMR transmission through dairy products.

## 5 Conclusion

This study reported the key issues of microbial contamination and AMR characteristics in cow bulk tank milk from dairy farms in Shandong Province, China. The combination of the antibiotic resistance status of the strains and the regional differences observed in farm practices further emphasizes the importance of rational antibiotic use to help alleviate the selection pressure for antibiotic resistance. At the same time, optimizing environmental hygiene management remains crucial for reducing the risk of bacterial transmission and the transfer of antibiotic resistance genes in the farm environment.

## Data Availability

The original contributions presented in the study are included in the article/supplementary material, further inquiries can be directed to the corresponding authors.
